# Therapeutic Effect of Berberine on Huntington’s Disease Transgenic Mouse Model

**DOI:** 10.1371/journal.pone.0134142

**Published:** 2015-07-30

**Authors:** Wenxiao Jiang, Wenjie Wei, Marta A. Gaertig, Shihua Li, Xiao-Jiang Li

**Affiliations:** 1 Department of Human Genetics, Emory University School of Medicine, 615 Michael Street, Atlanta, GA 30322, United States of America; 2 Department of Neurology, Tongji Hospital, Tongji Medical College, Huazhong University of Science and Technology, Wuhan 430032, China; 3 Graduate Program of Microbiology and Molecular Genetics, Emory University, Atlanta, GA 30322, United States of America; National Institute of Health, UNITED STATES

## Abstract

Huntington disease (HD) represents a family of neurodegenerative diseases that are caused by misfolded proteins. The misfolded proteins accumulate in the affected brain regions in an age-dependent manner to cause late-onset neurodegeneration. Transgenic mouse models expressing the HD protein, huntingtin, have been widely used to identify therapeutics that may retard disease progression. Here we report that Berberine (BBR), an organic small molecule isolated from plants, has protective effects on transgenic HD (N171-82Q) mice. We found that BBR can reduce the accumulation of mutant huntingtin in cultured cells. More importantly, when given orally, BBR could effectively alleviate motor dysfunction and prolong the survival of transgenic N171-82Q HD mice. We found that BBR could promote the degradation of mutant huntingtin by enhancing autophagic function. Since BBR is an orally-taken drug that has been safely used to treat a number of diseases, our findings suggest that BBR can be tested on different HD animal models and HD patients to further evaluate its therapeutic effects.

## Introduction

Huntington’s disease (HD) is a severe neurodegenerative disease that is characterized by chorea, dystonia, motor coordination loss, and mental deterioration. Age of HD onset is usually mid to late life, but in rare cases, may be seen in juveniles. The HD gene encodes huntingtin (Htt), a 350kDa protein with a variable-length polyglutamine (polyQ) tract encoded in exon 1 of the HD gene [1]. Expansion of the polyQ repeat tract (>36Q) results in HD, and increases of over 55Q lead to fast progression juvenile-onset HD [2, 3]. The reason for this threshold is most likely that expanded polyQ repeats cause N-terminal Htt fragments to misfold, leading to abnormal protein interactions and aggregation of mutant Htt [4, 5]. Although the role of Htt aggregates in HD remains controversial, they result from the accumulation of mutant Htt and have been used to assess the therapeutic effects of drugs on HD.

Currently, there is no effective treatment for HD, though some drugs, such as tetrabenazine and haloperidol, have been used in clinical studies for controlling symptoms of HD [6, 7]. One candidate that has shown promise through relatively recent discoveries is the plant-derived protoberberine alkaloid called Berberine (BBR). This small molecule with a molecular weight of 336.4 g/mol is derived from the roots and bark of various plants, such as *Coptis chinenses*, *Berberis sp*., and has been used for over six decades in Chinese medicine as an over-the-counter for treatment of bacterial diarrhea [8, 9]. However, recent discoveries have unearthed a bounty of additional uses for this botanical compound, including its ability to combat hypercholesterolemia, diabetes, cardiac disease, inflammation, and the side-effects of radiotherapy [10–17]. Due to the high tolerance for orally-taken doses (LD50>5g/kg), BBR has been proven safe for long-term use, is readily available in the bloodstream 2 hours after oral intake and, more importantly, is able to freely cross the blood-brain-barrier [14, 18, 19], which make it an ideal drug candidate to test its protective effects on chronic neurological disorders.

The most encouraging and relevant discovery to HD is BBR’s ability to ameliorate the symptoms and pathology of Alzheimer’s Disease (AD) and Parkinson’s disease (PD) animal models [16, 20–23]. The ability of BBR to reduce β-amyloid aggregation and accumulation in AD mice bears promising hope that it could do the same against HD, as both diseases are caused by the accumulation of misfolded proteins. Here, we examined the effects of BBR on mutant Htt’s accumulation and toxicity in cellular and animal models of HD. Our findings show that BBR is protective for HD transgenic mice and suggest that it can be safely tested in HD patients to evaluate its potential beneficial effects on HD symptoms.

## Materials and Methods

### Ethics statement

All procedures were performed in accordance with the NIH and U.S. Public Health Service’s Guide for Care and Use of Laboratory Animals and were approved by the Institutional Animal Care and Use Committee at Emory University with approved IACUC protocol (2002557), which is accredited by the American Association for Accreditation of Laboratory Care (AAALC). All of the care for the animals is consistent with standard operating procedures, and all efforts were made to minimize suffering.

### Mice

The N171-82Q mice were obtained from The Jackson Laboratory (strain 003627), which are a transgenic HD mouse model expressing 171 amino acids of the N-terminal Htt containing 82 glutamine repeats in the PolyQ tract under the mouse prion promoter. Male N171-82Q mice were mated with wild type female mice to produce offspring mice on a B6 x C3H background. Genotyping was performed using PCR on DNA obtained from tail samples. Primers for genotyping N171 82Q mice are as follows: sense S26: 5’-CTA CGA GTC CCT CAA GTC CTT CCA GC-3’, antisense A151: 5’-GAC GCA GCA GCG GCT GTG CCT G-3’. Wild type littermates and N171-82Q mice were used for investigation of their behaviors and the expression of transgenic mutant Htt. N171-82Q mice become increasingly symptomatic from 6–8 weeks with a short lifespan of 16–22 weeks. All mice were housed in groups of 4 in cages that measured 6.5” wide, 12” long, and 6” deep (Super Mouse Micro-Isolator, Patent Number 6,227,146) under a 12 h light/dark cycle with ad libitum access to Purina rodent chow and water at normal room temperature (22–25°C) and humidity. Corn husk bedding and 2 small bedding pads (2”x2” expandable into fluff upon chewing and tearing) were provided to each cage. Food pellets and autoclaved water were suspended from a feeding cage and drinking bottle; after 3 months age, pellets were also provided on the ground. Air was filtered on the top of the cage and machine-regulated. 4 groups were created: mutant group treated with BBR, mutant group treated with vehicle, WT group treated with BBR, and WT group treat with vehicle. Each group consisted of 7 animals: 5 males and 2 females. Gender-even groups were constructed to prohibit gender-induced differences in physical performance from influencing results of behavioral studies. Male and female mice were kept separately, and BBR and vehicle-treated mice were kept separately. The groups were created by separating the mutant and WT siblings of several litters all born within 4 days of each other. Sibling-matching into opposing groups was done whenever possible. The fathers of these litters were N171 82Q mutants from a single bloodline and the mothers were unrelated WT females.

### Antibodies and Reagents

Mouse monoclonal antibody to Htt (mEM48) is as described [24]. Rabbit anti-LC3B and mouse anti-β-actin were purchased from ABGENT; mouse anti-P62 was purchased from Sigma. Green fluorescent Alexa488 donkey anti-mouse and all secondary antibodies were purchased from Jackson Labs.

Berberine-chloride and BFA (Bafilomycin-A) were purchased from Sigma. Htt plasmids expressing exon1 Htt containing 120Q (HD120Q-GFP) or 20Q (HD20Q-GFP), which is fused with GFP, were generated in our previous studies [25].

### Cell culture and transfection

HEK293 cells were cultured in 12-well plates in DMEM/F12 (Invitrogen) containing 10% (vol/vol) FBS, 100 U/mL penicillin, 100 μg/mL streptomycin (Invitrogen), and 250 μg/μL Fungizone amphotericin B. Cells were maintained at 37°C in 5% CO_2_ incubators. At roughly 50% confluence, cells were transfected with 0.25 μg of plasmid DNA per well using Lipofectamine 2000 (2mg/mL from Invitrogen) for 6 h in serum free medium. Within the next 24–48 h, the following were added: BBR (dissolved in methanol to final concentration of 5–100 μM), BFA (dissolved in DMSO to final concentration of 200 nM). In each case, the dilution was made such that 1 μL of the dissolved BBR or BFA was added to the well with 1 mL culture media. One μL of the vehicle was added to the control wells. The cells were then harvested at 48 h after BBR treatment or 24 h after BFA treatment.

### Oral gavage of BBR

Four mg BBR were dissolved in 1 mL distilled H_2_O (dH_2_O sterilized and distilled purchased from Invitrogen) and mixed in a 2 mL tissue emulsifier (Wheaton Biopharmaceutical and Life Sciences Products, Millville, NJ). The solution of BBR (40 mg per kg or 10μl per gram of body weight) was gavaged daily to each mouse (without anesthesia due to the dangers of daily anesthesia and the need for alert feedback from the mouse in case of abnormal occurrence). The same volume of dH_2_O was gavaged into control mouse groups. Oral gavage was performed with 24G-1” 1.25 mm ball-tip stainless steel feeding needles slightly bent inserted along the back of the throat in mice. One mL syringes were used for the injection of BBR solution, which was done slowly without excessive pressure. If any mouse struggling was noticed, needle was withdrawn, and the mouse was given a brief rest before the needle was reinserted to administer the remaining solution. Mice were observed through the cage for roughly 15 minutes post-gavage to ensure normal and alert behavior. In case of morbid mice, they would be examined roughly 3 hours before planned gavage time, by handling and close examination and gavage was only given if they were determined to be strong enough to receive the gavage with the stress it entails. If not, they were sacrificed and brain tissue collected. Oral administration of BBR started from 4 weeks of age until the death or sacrifice of animals.

### Behavioral studies

To eliminate environmental biases and to assess the neurological symptoms of HD mice, we did not provide specific care to HD mice, which were maintained under the same housing conditions as control mice. Behavioral tests were performed in the early afternoon, at the same time for all groups, in a room that was set to minimize external sound or visual disturbance. Rotarod performance was examined using Rotamex from Columbus Instruments. Bottom position of drop pan was well-padded with tissue paper to diminish chance of injury from fall. Mice were first trained once a day for 3 consecutive days, allowing them to run freely for 10 min per training session. After training, each mouse was run in triplicate per test and the average was taken. If any value presented at less than half of other values, a retrial was given and counted only if it was higher than the initial value.

Grip strength was tested using Chatillon-Ametek 2lbf force meter mounted to Columbus Instruments grip force meter stand. Mice were allowed to grip the grid with all 4 appendages and were pulled with a single constant force by the tail until removed from the machine. Each mouse was tested in triplicate and the average was taken.

Balance beam test was run using a 0.6 cm thick meter stick suspended from a platform on both sides by metal grips. The total running distance was roughly 0.8 m. There is a bright light at the starting point and a dark box at the endpoint. Prior to data-collection, each mouse was trained for 3 consecutive days with 3 runs per day. After training, mice were allowed to run only 1 time, since consecutive runs always yielded increasingly inferior results due to lack of will/curiosity, and the time taken to cross the beam was collected.

### Sacrifice

The bodyweights of mice were at first monitored weekly, but increased to bi-weekly near the final weeks of the experiment as the mice atrophy. Mice were monitored daily before each gavage for excessive trembling, loss of coordination and muscle weakness. The endpoint was reached when a sudden weight drop of ~20% over a week was coupled with severe tremor, weakness, or abnormal gait. When the mice reached this point, they were sacrificed for their brain tissue. They would be put into an isofluorane chamber until recumbent, limp, and unresponsive to stimuli such as toe-pinching, then swiftly decapitated with a scissor and the brains harvested with one half stored in an 1.5ml Eppendorf tube at -80°C and the other half preserved in Tissue Plus Optimum Cutting Temperature Compound (Fisher Healthcare) and stored at -80°C as well. However, despite these efforts, 6 out of 14 mutant mice died of disease without being sacrificed. (There were no unexpected losses in WT mice.) In these cases, they died without reaching the described endpoint as sometimes N171 82Q mice may pass without obvious debilitation. Care was taken not to over-assess symptoms and sacrifice too early in order to provide more accurate survival data.

### Fluorescent microscopy

Cultured cells were fixed with 4% paraformaldehyde (Electron Microscopy Sciences) for 10 min at room temperature and then treated with 0.5% saponin (Fluka). They were then stained with 1:3,000 Hoechst solution for nuclear labeling. The plates were then used directly for fluorescent microscopy to examine the expression of Htt-120Q-GFP and Htt-20Q-GFP.

For immunostaining of mouse brain sections, the freshly-isolated brain tissues were preserved in Tissue Plus Optimum Cutting Temperature Compound and transferred from -80°C for cutting to 10 micron-slices with the Leica CM1850 cryo-cutting machine set at -20°C. The sections were mounted onto slides and preserved in 4% paraformaldehyde for 10 min and washed 3 times in PBS before blocking in 3% bovine serum albumin (BSA), 0.3% Triton X-100 for 2 h at room temperature. Primary antibody mEM48 in 3% BSA was incubated with brain sections for 16 h at 4°C. After washing in PBS, the secondary antibody (green fluorescent Alexa488 donkey anti-mouse) was added at 1:5,000 for incubation at 4°C for 1–2 h. The slides were then washed again in PBS 3 times before microscopic examination.

### Western Blotting

Cultured cells (in 12-well plates) were dissolved in SDS-1% Triton 100-X /PBS at 125 μL per well. The cells were collected into Eppendorf tubes and sonicated (QSonica, LLC XL-2000 at setting 1) for 10 sec on ice. After boiling for 10 min, the samples were loaded into a 4–20% polyacrylamide SDS gel. When brain tissues were analyzed, homogenization buffer was first made freshly by adding 1% Triton 100X, protease inhibitor Pierce 78430 1:100, phenylmethanesulfonyl fluoride 100 μm/mg (Sigma), 2 mM sodium orthovanadate 1:200 (Sigma), and sodium fluoride 100 μM (Sigma) to PBS. The mouse brain cortex was homogenized in 100 volumes of the chilled homogenization buffer. The tissue extracts were then sonicated for 10 sec on ice and rocked (Boekel Scientific Orbitron Rotator II Model 260250 at max rpm) for 30 min at 4°C before being spun down at 16,000 RPM for 15 min at 4°C. The supernatant was then taken and combined with 0.25 volumes 5x loading dye, followed by boiling for 10 min and sonicated again for 5 sec. The samples were resolved by SDS gel, which was transferred to a nitrocellulose membrane (GE Healthcare Life Science) for 1 hr, which was then blocked for 1 hr in 5% Nestle/Carnation brand powdered nonfat bovine milk in PBS. After washing 3 times in PBS for 10 min, each primary antibody in 3% BSA was used to probe the blots for 16 h at 4°C while rocking. Secondary antibodies (donkey anti-mouse, goat anti-rabbit) were used at 1:5,000 for 2 h while rocking at room temperature, and the HyGLO QuickSpray kit (Denville Scientific) and the Konica Minolta SRX-101A film developer were used to detect immunostaining signals.

### RT-PCR

Total RNA was isolated from the brain cortex tissues of 3 BBR-treated N171-82Q transgenic mice and 3 control mice using the RNeasy Lipid Tissue Mini Kit (Qiagen). Reverse transcription reactions were performed with 1.5 μg of total RNA using the Superscript III First-Strand Synthesis System (Invitrogen, 18080–051). cDNA (100 ng) was combined with 10 μl SYBR Select Master Mix (Applied Biosystems, 4472908) and 1 μl of each primer in a 20-μl reaction. The reaction was performed in the Eppendorf, Realplex Mastercycler thermocycler. The sequences of the primers are as follows:

Htt sense: 5′-ATGGCGACCCTGGAAAAGCT-3’; Htt antisense: 5-TGCTGCTGGAAGGACTTGAG-3′.

GAPDH sense: 5’-AACTTTGTCAAGCTCATTTCCTGGT-3’; GAPDH antisense: 5’-GGTTTCTTACTCCTTGGAGGCCATG-3’

### Statistical analysis

Data collection (other than on the balance beam) was done using the average of three trials as the final score for the animal on that day. If, of the three scores, one fell below 50% of the average of the other two, a re-test was given to prevent isolated incidents from greatly affecting the overall score. If the retest results were still below the 50% mark, the higher of the two lowest values (retested score vs. original lowest score) was taken into the mean calculations.

Differences between two groups were evaluated by 2-tailed Student's t-test. For behavioral analysis, 7 mice per group were examined, and the results were expressed as the mean± SEM. For *in vitro* experiments, at least three independent experiments were performed to obtain the data (mean± SEM). A P-value of <0.05 was considered significant.

## Results

### BBR reduces Htt aggregation in transfected HEK293 cells

**Fig 1:**
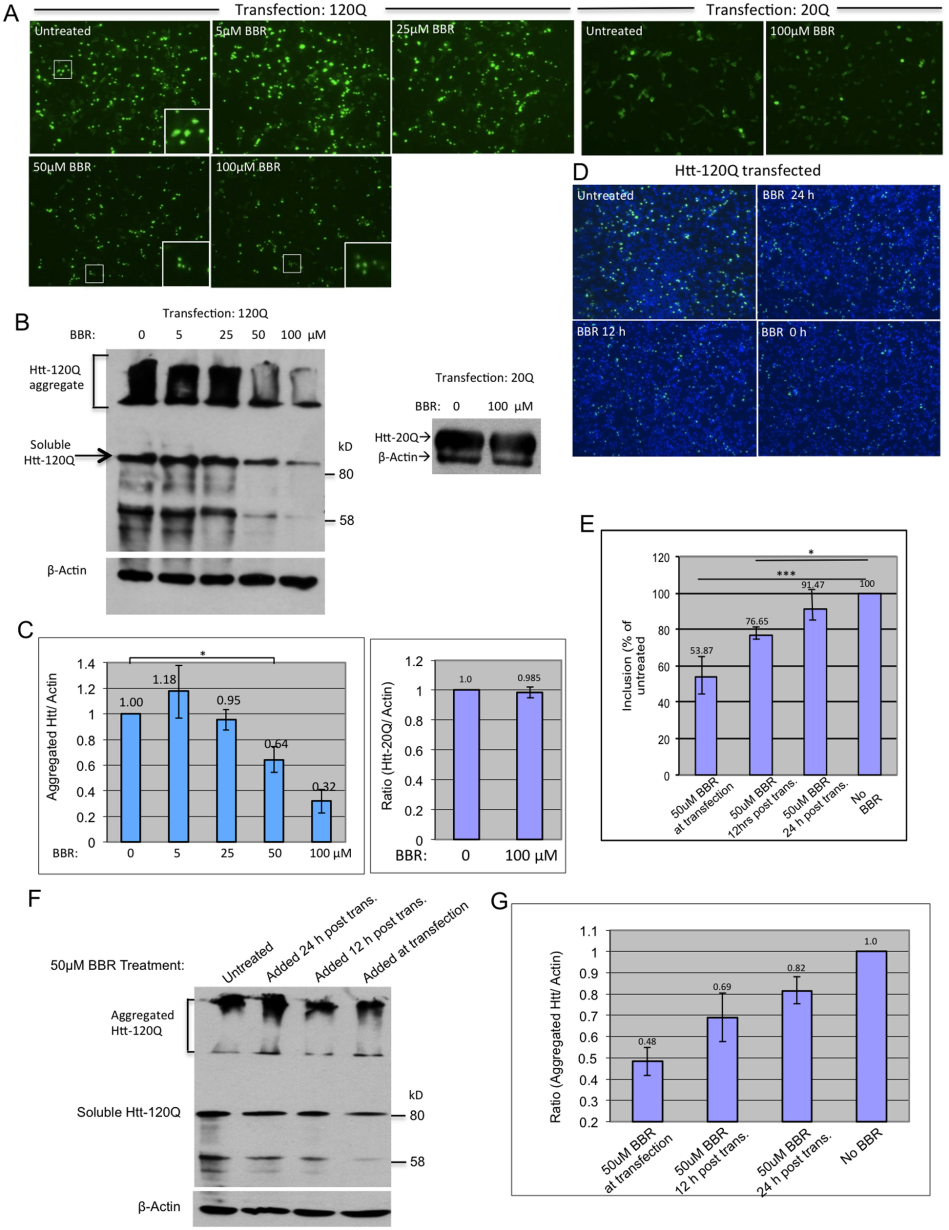
BBR reduced Htt aggregation *in vitro* in a dose and time-dependent manner. **(A)** Immunocytostaining images (10 X) of Htt-120Q- or Htt-20Q-transfected HEK293 cells that were treated with different concentrations of BBR (0, 5, 25, 50, 100 μM). **(B)** Western blot analysis of Htt-transfected cells treated with or without BBR at different concentrations. Aggregated Htt in the stacking gel was detected by mEM48 antibody. Soluble mutant Htt and its potential degraded products are also shown. **(C)** Densitometry analysis of the ratios of aggregated Htt or Htt-20Q to β-actin on western blots in (B). **(D)** Fluorescent microscopic images of Htt-120Q-transfected HEK293 cells that were treated with 50 μM BBR at 0 h, 12 h or 24 h post-transfection. **(E)** Cell-counting analysis of images obtained in (D) showing the percentage of aggregates relative to the total cells revealed by DAPI nuclear staining. **(F)** Western blotting of Htt-120Q transfected HEK293 cells treated with 50 μm BBR for different times showing aggregated Htt in the stacking gel. **(G)** Densitometry analysis of the ratios of mutant Htt to β-actin on western blots in (F). The quantitative data are presented as mean±SE.

First, we wanted to see whether BBR would produce any effect on the accumulation of mutant Htt in transfected cells. BBR was added to the culture medium at concentrations of 0, 5, 25, 50, and 100 μM immediately after transfection of HEK293 cells with GFP-exon1 Htt containing 120Q (Htt-120Q). After 48 h of incubation with BBR, the cells were examined via fluorescent microscopy. The results showed a dose-dependent decrease of Htt-120Q aggregates, which were presented as puncta, with notable reduction at 50 μM BBR (Fig 1A). However, HEK293 cells transfected with the control GFP-exon1 Htt containing 20Q (Htt-20Q) did not exhibit any significant GFP signal reduction with even the highest concentration of BBR (100 μM), suggesting that BBR selectively reduces the accumulation of mutant Htt (Fig 1B) and since both Htt-20Q and Htt-120Q were both under a cytomegalovirus promoter, it also suggests that BBR does not impede transfection or promoter activity. The effect of BBR on reducing Htt aggregation was also shown by Western blotting that revealed aggregated Htt in the stacking gel. Quantification of the ratios of aggregated Htt to actin via densitometry also verified the reduction of Htt aggregates by BBR (Fig 1B and 1C) (for treatment with 50uM BBR, P = 0.028, T = 5.85, DF = 2). These results suggest that BBR can suppress the aggregate formation or the accumulation of mutant Htt (Fig 1A–1C).

Mutant Htt aggregates can become stabilized after they are formed in cells. We wanted to see whether BBR could reduce mutant Htt aggregation after HEK293 cells had expressed mutant Htt for different times. Thus, 50 μm BBR was added to Htt-120Q transfected cells at 0, 12, or 24 h post transfection and then incubated with the cells for 48 hours. Immunofluorescent staining images showed time-dependent effects on Htt aggregates, with the greatest reduction of Htt aggregates when HEK293 cells were treated immediately with BBR after transfection and diminishing effect as the treatment was delayed. However, adding BBR at 12-hours post transfection still produced a pronounced inhibitory effect (Fig 1D). The percentage of cells containing Htt aggregates relative to total cell numbers, which were revealed by the nuclear DAPI staining, was also significantly reduced by BBR (Fig 1E) (for treatment 12-hours post transfection, P = 0.0008, T = 34.9, DF = 2). Western blotting analysis of aggregated proteins and quantification of the relative levels of aggregated Htt confirmed that the earlier BBR treatment yielded the greater reduction in Htt aggregation (Fig 1F and 1G) (for treatment 12-hours post transfection, P = 0.123, T = 2.58, DF = 2).

### BBR activates the autophagy pathway

The effect of BBR to reduce Htt aggregates led us to investigate whether BBR can increase the ability of cells to clear misfolded proteins. Since BBR has been found to up-regulate autophagy in cancer cells [26, 27], the autophagy pathway was selected as the primary candidate for investigation.

**Fig 2:**
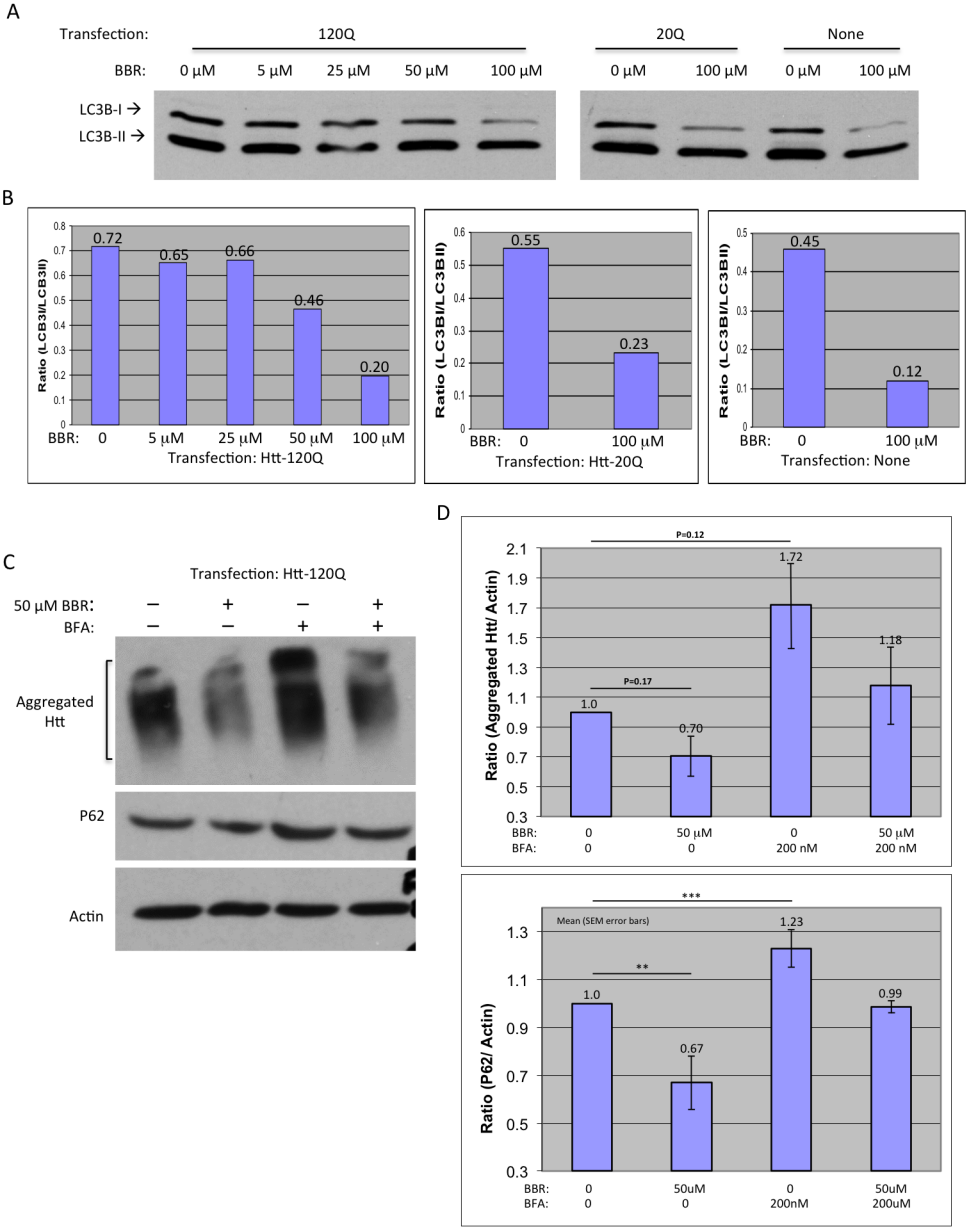
BBR increases autophagic activity in cultured cells. **(A)** Western blotting of LC3B in HEK293 cells transfected with or without Htt and treated with BBR (0, 5, 25, 50, or 100 μM). **(B)** Densitometry analysis of the ratios of LC3-I to LC3-II in above Western blots. **(C)** Western blotting of Htt-transfected HEK293 cells treated with or without 50 μM BBR or bafilomycin (BFA), an autophagy inhibitor. Antibody to P62, which decreases when autophagy activates, was used to confirm the altered activity of autophagy. **(D)** Densitometry analysis of the ratios of aggregated Htt or P62 to actin in Western blots in (C). The quantitative data are presented as mean±SE.

To determine autophagic flux, we examined LC3B, which converts from form I to from II to serve as the recruiter of autophagosome substrate P62 during the activation of autophagy. Non-transfected or Htt-transfected HEK293 cells were treated with 0, 5, 25, 50, or 100 μM BBR for 48 h. Western blotting analysis of LC3B-I and LC3B-II (Fig 2A) and densitometry were done to compare LC3-I/LC3-II ratio (Fig 2B). A lower ratio indicates more conversion of LC3-I to LC3-II or activation of autophagy. This ratio was decreased as the concentration of BBR was increased, suggesting that BBR could increase autophagic function (Fig 2B). Increased autophagic activity was also observed in both non-transfected cells and cells transfected with the control Htt-20Q (Fig 2B). To establish a causative relationship between autophagic increases and Htt aggregate reduction, we inhibited autophagy in Htt-120Q-transfected HEK293 cells using bafilomycin A (BFA) and treated these cells with 50 μM BBR. Western blotting showed that BBR reduced Htt aggregation and also antagonized the effect of BFA on increasing Htt aggregation (Fig 2C) (BBR only: P = 0.173, T = 2.08, DF = 2; BFA only: P = 0.124, T = 2.57, DF = 2). P62 was used as an autophagic indicator, as it is an expendable substrate that decreases with autophagic up-regulation. We also saw a decrease in P62 after BBR treatment and its increase after BFA treatment, verifying BFA’s activity to inhibit autophagy (BBR only: P = 0.008, T = 11.11, DF = 2; BFA only: P = 0.0008, T = 35.34, DF = 2). The opposite effects of BBR and BFA on Htt aggregation and P62 were further confirmed by densitometry quantifying of the Western blot results (Fig 2D).

### BBR ameliorates the neurological phenotypes in transgenic N171-82Q mice

Next, we wanted to see if the inhibitory effect of BBR on the accumulation of mutant Htt in cultured cells could be mirrored in an HD animal model and result in phenotypical improvements. The N171-82Q mice, which express N-terminal mutant Htt with 82Q and show progressive neurological phenotypes [28], were divided into BBR and vehicle-treated groups (n = 7 mice per group). These mice were orally gavaged daily with dH_2_O or 40 mg/kg BBR that was dissolved in dH_2_O. WT siblings were also set up into groups (n = 7 per group) treated with BBR or dH_2_O. BBR treatment was started at 4 weeks of age and continued until sacrifice or death of mice, and mouse behaviors were tested at 6 weeks of age.

**Fig 3:**
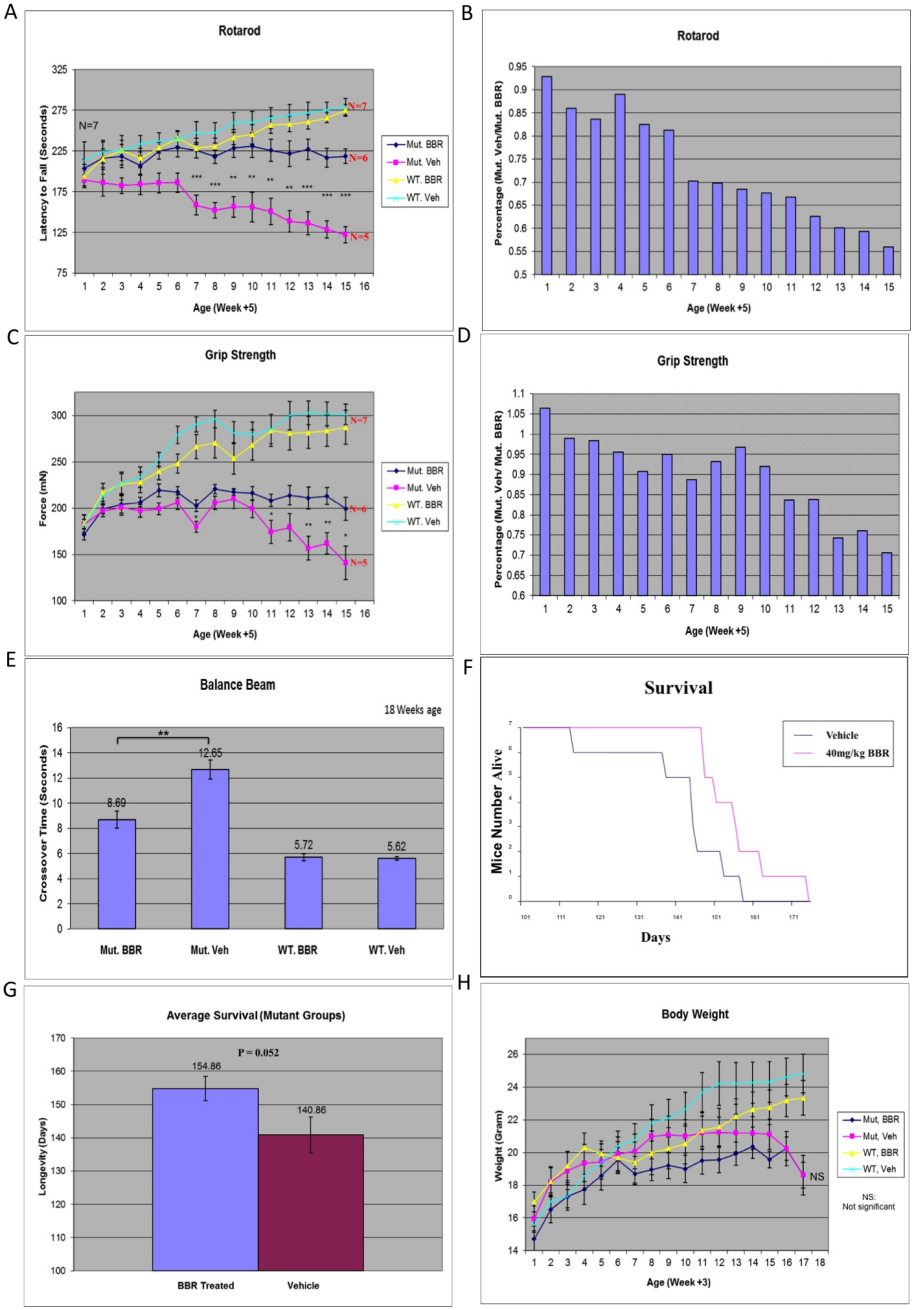
Oral administration of BBR ameliorates neurological symptoms in transgenic N171-82Q mice. **(A, B)** Rotarod test of motor coordination of N171-82Q transgenic mice and WT siblings, orally gavaged with 40 mg/kg BBR or vehicle daily. **(C, D)** Grip strength test on transgenic N171-82Q mice and WT siblings treated with BBR or vehicle. **(E)** Balance beam test on 18-week-old N171-82Q and WT mice that had been treated with BBR or vehicle. **(F)** Survival curve for N171-82Q mice and WT siblings treated with BBR or vehicle. (**G**) The BBR-treated N171-82Q group survived 15 days longer than their untreated HD siblings. **(H)** Body weight of N171-82Q mice and WT siblings treated with or without BBR. The quantitative data are presented as mean±SE. *p<0.05. **p<0.01. ***p<0.001, n = 7 mice per group.

The rotarod performance was one of the key tests for assessing motor coordination in a variety of HD mouse models. Rotarod tests, which were conducted weekly from week 6 to 21, indicated that HD mice treated with BBR showed significant improvements on their rotarod performance as compared to HD siblings without BBR treatment (P = 0.0002, T = 9.68, DF = 5) (Fig 3A). When comparing the rotarod test scores of the BBR-treated and untreated HD mice, one can see an almost linear relative decline of rotarod performance for the untreated group as compared to the BBR treated group (Fig 3B).

Muscular weakness is another key feature of HD mice, which can be assessed by the grip strength test. Grip strength tests conducted weekly from week 6 to 21 also indicated that the average muscle strength of HD mice was markedly increased after BBR treatment as compared with untreated HD siblings (P = 0.0057, T = 4.63, DF = 5) (Fig 3C and 3D). The difference between the untreated and treated HD mice in grip strength became obvious after the poor rotarod performance became apparent (Fig 3A and 3C), suggesting that muscular weakness followed neuronal dysfunction in HD mice.

We also used the balance beam test to examine motor coordination function and balance. HD mice were given a balance beam assessment in which the mice walked across a beam (Fig 3E). The shorter time for the mouse to successfully get across the beam is correlated with the greater motor coordination and balance. The results showed that while BBR-treated HD mice did not exhibit the same dexterity as WT siblings, they were faster than their untreated HD siblings (mean values: 8.69 s for BBR-treated, and 12.65s for untreated HD mice, P = 0.0025, T = 5.60, DF = 5) (Fig 3E).

Life span is another critical criterion for evaluating treatment efficacy, and we also saw that BBR improved the life span of N171-82Q mice. In general, untreated HD mice began to die before their BBR-treated siblings (life span range: untreated HD mice = 112–156 days; BBR-treated HD mice = 146–173 days) (Fig 3F). The average life span was 140.86 days for the untreated HD mice and 154.86 days for the BBR-treated HD mice, which is roughly a 10% difference (Fig 3G) (P = 0.052, T = 2.42, DF = 6). Despite their prolonged life span, BBR-treated HD mice did not show any significant alteration in body weight compared with the untreated HD group (P = 0.989, T = 0.014, DF = 5) (Fig 3H).

### BBR treatment reduced Htt aggregation and increased autophagy in HD mice

**Fig 4:**
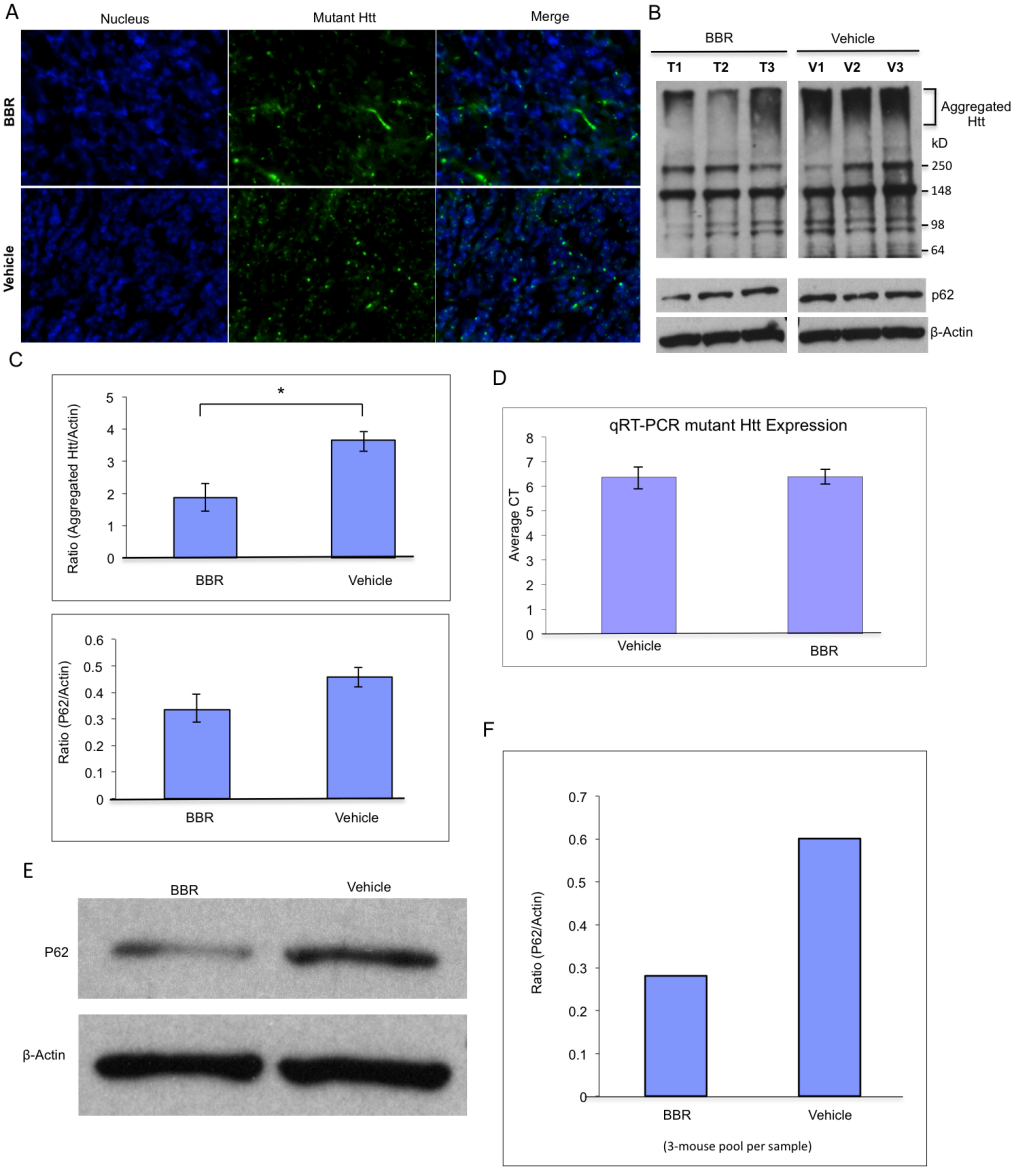
Oral administration of BBR reduced mutant Htt aggregation and increased autophagy in transgenic N171-82Q mice. **(A)** Fluorescent immunohistostaining of mouse brain slices from transgenic N171-82Q HD mice and WT siblings, orally gavaged with 40 mg/kg BBR or vehicle daily. **(B)** Western blotting of the cortex samples of 3 mice per group (HD+BBR vs. HD+vehicle) showing mutant Htt aggregates in the stacking gel and P62. Non-specific immunoreactive bands are also shown on the blots. Actin was used to show the internal control protein on western blot. **(C)** Densitometry analysis of above Western blots. The data are mean±SE (n = 3). **(D)** Quantitative RT-PCR analysis of transgenic mutant Htt mRNA in the cortex of the BBR-treated or untreated N171-82Q mice. n = 3 mice per group. Data are presented as mean CT±SEM. **(E)** Western blotting of WT mouse cortex samples (3-mouse pool per group) after the mice had been orally gavaged with 40 mg/kg BBR or vehicle daily for 24 weeks and sacrificed 4 h after the last BBR administration. **(F)** Densitometry analysis of Western blots in (E) showing the ratio of P62 to actin. The quantitative data are presented as mean±SE.

Based on the results from cultured cells, we wanted to examine whether BBR also reduced mutant Htt accumulation and aggregation in the HD mouse brain. Immunofluorescent staining of cerebellar brain slices revealed a drastic reduction of mutant Htt aggregates in the BBR-treated HD mouse brains as compared to the untreated HD mouse brains (Fig 4A). Western blotting of brain cortex samples from 3 separate mice per group was done and quantified by densitometry, revealing nearly 50% reduction in aggregated Htt in the BBR-treated HD mice as compared to their untreated HD siblings (Fig 4B and 4C) (P = 0.03, T = 5.64, DF = 2). However, the antibody (mEM48) used is poorly suited for detecting soluble mutant Htt in animal tissue despite the presence of non-specific immunoreactive bands (Fig 4B). To investigate whether this reduction occurred at the transcriptional level, RT-qPCR was performed on the HD mouse brain samples that were treated with or without BBR. The results show that BBR did not alter the transcriptional levels of transgenic mutant Htt (Fig 4D) (P = 0.988, T = 0.017, DF = 2). Thus, BBR is likely to inhibit the accumulation of mutant Htt at the protein level. To examine whether BBR stimulates autophagy to clear mutant Htt, we also probed the western blots with an antibody to P62 and found a moderate decrease in P62 in the BBR-treated group (Fig 4B and 4C) (P = 0.06, T = 3.90, DF = 2). Because the BBR-treated HD mice were examined for their behaviors and then sacrificed when they were near death, the most pronounced effects of BBR on autophagy in the HD mouse brain might not have been detected. Thus, the WT control group, which was gavaged with BBR daily at the same time as their HD siblings, was given their last gavage and sacrificed 4 hours later. The brain cortex samples of BBR-treated and untreated mice were then collected for western blot analysis with anti-P62. Densitometry analysis revealed a much more marked decrease in P62 in the BBR-treated mouse brain (Fig 4E and 4F), which also supports the idea that BBR treatment can increase autophagic activity in the mouse brain.

## Discussion

HD shares many pathological features with other neurodegenerative diseases that are caused by protein misfolding. These pathological features include age-dependent accumulation of misfolded proteins and selective degeneration of neuronal cells. Since HD is a monogenetic disorder caused by polyQ expansion in Htt, HD provides an ideal model for us to find therapeutics for neurodegenerative diseases. However, there is still lack of effective treatments for HD, despite large efforts being made to identify its therapeutics [29, 30].

In the current study, we found that BBR can effectively improve motor function of N171-82Q mice. This improvement is evident by the significant increase in performance on the rotarod test by N171-82Q mice after BBR treatment. The increased grip strength of HD mice by BBR treatment also indicates the alleviation of muscle weakness caused by mutant Htt. Also, balance beam test results support the improved motor function and movement coordination of HD mice after BBR treatment. All these effects could contribute to the prolonged life span of BBR-treated HD mice. We also observed that BBR did not seem to prevent loss of bodyweight (Fig 3F). This may be because N171-82Q mice have metabolic defects mediated by mutant Htt in both CNS and peripheral tissues [31, 32] and BBR may induce weight loss via cholesterol reduction [9] so this effect may mask any rescuing effects on body weight. The dosage of BBR used in this study was 40mg/kg. We also tried two higher doses (150 and 250mg/kg) but both did not yield greater effects than the dosage of 40mg/kg. It remains to be seen if other doses of BBR would produce a greater protective effect on HD mouse symptoms.

BBR has been found to have protective effects on a wide range of pathological events [10–17]. Such diverse effects could account for the protective effect of BBR on HD mice. How BBR can mediate broad beneficial effects against different pathological events remains unknown. It is possible that BBR may act on some common pathological pathways shared by many disease conditions. With potential effects against neurodegenerative diseases, BBR has also exhibited several known effects such as inhibition of monoamine oxidase B (MAO-B), acetylcholinesterase (AChE), up-regulation of nuclear factor erythroid 2 (Nrf2), (glycagon-like protein 1) GLP-1, as well as phosphorylation of Protein kinase B (AKT) and CREB [33–37]. Inhibition of MAO-B and up-regulation of Nrf2 may help to defend against damage from reactive oxygen species (ROS) while inhibition of AChE as well as activation of phosphoinositide 3-kinase (PI3K)-AKT pathways defend against apoptosis [38–40]. GLP-1 plays a role in preservation of dopaminergic neurons and phosphorylated cyclic adenosine monophosphate response element-binding protein (CREB) may be related to the ability of neurons to survive damage [41–44]. Our findings suggest that BBR can activate autophagic function to reduce the accumulation and aggregation of mutant Htt. Although BBR decreases the level of both aggregate and soluble overexpressed mutant htt in transfected cells, it does not affect the level of transfected normal Htt. Furthermore, RT-PCR indicates that BBR does not influence the transcriptional expression of mutant htt, though it can reduce the formation of Htt aggregates in the HD mouse brains. Thus, decreasing mutant Htt accumulation at least contributes to BBR’s therapeutic effect and may be mediated by increasing autophagic function. This therapeutic effect is different from the previous findings for other therapeutics, which rely on improving transcriptional function [45], metabolic function [46, 47], neurotrophic factor signaling pathways [48], mutant Htt conformation, and its interactions with other proteins [24, 49]. The increased autophagic function by BBR could also be beneficial for other pathological conditions in which the accumulation of misfolded proteins leads to neurodegeneration. Consistent with this idea, BBR has been found to reduce the neuropathology in AD transgenic mice [16, 20– 23]. It is also known that the accumulation of toxic forms of peptides and misfolded proteins can lead to a variety of pathological events such as inflammation and altered cellular signaling [50, 51], which could explain the broad protective effects of BBR on a variety of pathological events.

Although our findings suggest that BBR may increase autophagic function to reduce mutant Htt accumulation and the associated neurological phenotypes in HD mice, it remains to be investigated how BBR can up-regulate the autophagy function. Such investigation would require more in-depth studies with sophisticated tools that can explore whether BBR directly associates with autophagic proteins to modulate their function. Despite this, there is a need to report the beneficial effects of BBR on HD, because BBR is an over-the-counter drug that has been safely used to treat a number of diseases. Discovery of its protective effects in N171-82Q mice may promote further exploration of the use of BBR in other HD mouse models as well as patients and perhaps other neurodegenerative diseases that are also caused by misfolded proteins.
